# Longitudinal genomic analyses of automatically-recorded vaginal temperature in lactating sows under heat stress conditions based on random regression models

**DOI:** 10.1186/s12711-023-00868-1

**Published:** 2023-12-21

**Authors:** Hui Wen, Jay S. Johnson, Pedro H. F. Freitas, Jacob M. Maskal, Leonardo S. Gloria, Andre C. Araujo, Victor B. Pedrosa, Francesco Tiezzi, Christian Maltecca, Yijian Huang, Allan P. Schinckel, Luiz F. Brito

**Affiliations:** 1 Department of Animal Sciences, Purdue University, West Lafayette, IN USA; 2 USDA-ARS Livestock Behavior Research Unit, West Lafayette, IN USA; 3 Department of Animal Science, North Carolina State University, Raleigh, NC USA; 4 Department of Agriculture, Food, Environment and Forestry, University of Florence, Florence, Italy; 5 Smithfield Premium Genetics, Rose Hill, NC USA

## Abstract

**Background:**

Automatic and continuous recording of vaginal temperature (T_V_) using wearable sensors causes minimal disruptions to animal behavior and can generate data that enable the evaluation of temporal body temperature variation under heat stress (HS) conditions. However, the genetic basis of T_V_ in lactating sows from a longitudinal perspective is still unknown. The objectives of this study were to define statistical models and estimate genetic parameters for T_V_ in lactating sows using random regression models, and identify genomic regions and candidate genes associated with HS indicators derived from automatically-recorded T_V_.

**Results:**

Heritability estimates for T_V_ ranged from 0.14 to 0.20 over time (throughout the day and measurement period) and from 0.09 to 0.18 along environmental gradients (EG, − 3.5 to 2.2, which correspond to dew point values from 14.87 to 28.19 ˚C). Repeatability estimates of T_V_ over time and along EG ranged from 0.57 to 0.66 and from 0.54 to 0.77, respectively. T_V_ measured from 12h00 to 16h00 had moderately high estimates of heritability (0.20) and repeatability (0.64), indicating that this period might be the most suitable for recording T_V_ for genetic selection purposes. Significant genotype-by-environment interactions (GxE) were observed and the moderately high estimates of genetic correlations between pairs of extreme EG indicate potential re-ranking of selection candidates across EG. Two important genomic regions on chromosomes 10 (59.370–59.998 Mb) and16 (21.548–21.966 Mb) were identified. These regions harbor the genes *CDC123*, *CAMK1d*, *SEC61A2*, and *NUDT5* that are associated with immunity, protein transport, and energy metabolism. Across the four time-periods, respectively 12, 13, 16, and 10 associated genomic regions across 14 chromosomes were identified for T_V_. For the three EG classes, respectively 18, 15, and 14 associated genomic windows were identified for T_V_, respectively. Each time-period and EG class had uniquely enriched genes with identified specific biological functions, including regulation of the nervous system, metabolism and hormone production.

**Conclusions:**

T_V_ is a heritable trait with substantial additive genetic variation and represents a promising indicator trait to select pigs for improved heat tolerance. Moderate GxE for T_V_ exist, indicating potential re-ranking of selection candidates across EG. T_V_ is a highly polygenic trait regulated by a complex interplay of physiological, cellular and behavioral mechanisms.

**Supplementary Information:**

The online version contains supplementary material available at 10.1186/s12711-023-00868-1.

## Background

Heat stress (HS), which occurs when an individual cannot dissipate body heat adequately to maintain thermal equilibrium under hot conditions, is a common problem in livestock production [1]. It compromises animal welfare and causes significant economic losses in animal production, reproductive performance, and animal health [2–4]. Furthermore, intensive genetic selection for a limited number of performance traits, such as litter size and milk yield (through litter weight at weaning), has contributed to increased metabolic heat production in modern sows and may have led to their greater sensitivity to environmental conditions [5]. Global warming further aggravates these effects and highlights the need for selecting more climatic resilient animals. Over the past decades, numerous studies have investigated the underlying mechanisms of the response to HS in various species [6–11]. Dozens of genes and quantitative trait loci (QTL) associated with response to HS have been identified [12, 13].

Measurements of core body temperature have been widely used and are considered reliable indicators for HS in livestock [14]. Unlike other core body temperature indicators (e.g., rectal temperature), automatically-measured vaginal temperature (T_V_) reduces disruptions in animal behavior, captures diurnal changes in body temperature, and allows continuous data to be recorded [15, 16]. A sensitive HS indicator that is not affected by disturbances from animal behaviors can play an important role in breeding programs, especially for lactating animals that are more heat-sensitive due to greater metabolic heat production compared to non-lactating sows [17]. However, little is known about the genetics of continuously-monitored T_V_ (i.e., longitudinal data) as a HS indicator in lactating sows.

Random regression models (RRM) have been used in breeding to investigate longitudinal data, as they can provide more precise results over time and across environmental gradients (EG) than other methods [18, 19]. Legendre orthogonal polynomials (LEG) are common functions that are used to model the fixed and random trajectories for longitudinal traits. However, B-spline functions (BS) can provide a similar fit to the data without having implausible estimates at the extremes of the trajectory curves that often result when fitting high-order polynomials [20]. However, fitting too many BS parameters can result in convergence problems and high computational requirements.

Gene expression data from broiler small intestine tissue and *caenorhabditis elegans* has been shown to exhibit a temporal dynamic profile during HS conditions [21, 22], since some genes have a greater role in specific regulatory functions and stages of HS response or recovery. This suggests that the effects of HS-related genes may vary with time or with environmental conditions. In addition, Oliveira et al. [23] showed that, when longitudinal traits are analyzed in dairy cattle (e.g., milk production and somatic cell score), differential sets of candidate genes are identified for different lactation stages.

Intra-vaginal sensors [24] enable automatic recording of Tv, which enables evaluation of the genetics of response to HS in lactating sows from a longitudinal perspective, and to identify genomic regions, candidate genes, biological processes, and metabolic pathways associated with this trait. Such analyses could contribute to understanding potential changes in the biological mechanisms that underlie functions involved in coping with HS and to optimize genetic progress for climatic resilience. Worldwide breeding programs are conducted on farms with considerably different environmental conditions and management choices [25], and can provide data to investigate the presence of significant genotype-by-environment interactions (GxE) for heat tolerance in lactating sows. Therefore, the main objectives of this study were to: (1) define statistical models and estimate genetic parameters for automatically-recorded Tv in lactating sows at different time points throughout the day and along EG; (2) assess potential GxE interactions for heat tolerance of lactating sows; and (3) identify genomic regions and biological processes that contribute to response to HS in maternal-line lactating sows from a longitudinal perspective.

## Methods

### Animals and datasets

All datasets used in this study were collected on a commercial farm in Maple Hill, NC, USA (34.70738° N, 77.73653° W) as previously described by Johnson et al. [24]. Sows were provided ad libitum access to feed and water. The T_V_ of 1381 sows from the studied population, which included 1645 lactating sows (parities 2 to 7; Landrace × Large White cross), was automatically measured every 10 min for 5 consecutive days between days 8 to 20 of lactation, as previously described [24]. In total, 932,681 T_V_ records were obtained. First parity sows were not included in this study because the farm where the experiment was conducted had only second and later parity sows (a common practice in some regions in North America). The ambient temperature (T_a_) and relative humidity (RH) of each room were recorded every five min [24], from which dew points were calculated using the Magnus–Tetens equation. Dew point is a thermal index that takes both temperature and humidity into account, and was chosen as environmental indicator due to its ability to accurately represent heat stress conditions, as previously demonstrated [30]. Outliers with environmental and T_V_ records that deviated by more than 3.5 standard deviation (SD) from the mean were discarded. T_V_ records below 37 °C were also deleted.

In total, 1639 sows (including all sows with phenotypic records) were genotyped using the PorcineSNP50K Bead Chip (50,703 SNPs; Illumina, San Diego, CA, USA). For quality control (QC) of the genotype data, single nucleotide polymorphisms (SNPs) with a minor allele frequency (MAF) higher than 0.01, or that had an extreme difference between observed and expected heterozygous frequencies less than 0.15, and SNPs and samples with a call rate greater than 0.90 were kept for further analyses. The QC was implemented using the BLUPF90 + family software [26] and 49,547 SNPs and 1639 animals remained for further analyses. A schematic representation of all analyses performed in the study is shown in Additional file 1: Fig. S1.

### Statistical models to estimate genetic parameters across time

Twelve single-trait RRM models were evaluated, with random regressions fitted as time-derived covariates using LEG (linear, quadratic, and cubic) and BS (linear, quadratic, and cubic with 5, 6, or 7 knots). The general formulation of the RRM used for the estimation of variance components and genome-wide association study (GWAS) is:

$${y}_{ijklmn}={Par}_{k}+{Loc}_{l}+{Date}_{m}+\sum_{r=1}^{R}{\gamma }_{ir}{\phi }_{r}\left({t}_{ij}\right)$$

$$+\sum_{r=1}^{R}{\alpha }_{ir}{\phi }_{r}\left({t}_{ij}\right)+\sum_{r=1}^{R}{\rho }_{ir}{\phi }_{r}\left({t}_{ij}\right)+{e}_{ijklmn},$$

where ${y}_{ijklmn}$ is the $n$th T_V_ observation, recorded at the $j$th time point of animal $i$ from the $k$th parity, at location $l$, and date of measurement $m$; ${Par}_{k}$ is the fixed effect of the $k$th parity of animal $i$; ${Loc}_{l}$ is the fixed effect of the $l$th location (e.g., barn and room within barn) of animal $i$; ${Date}_{m}$ is the fixed effect of the $m$th measurement date of animal $i$; ${\gamma }_{ir}$ is the $r$th fixed regression coefficient specific for the time trajectory; ${\alpha }_{ir}$ and ${\rho }_{ir}$ are the $r$th random regression coefficients that describe the trajectory of the additive genetic effects and permanent environmental effects of animal $i$, which were assumed to follow ${\varvec{\upalpha}}\sim {\text{N}}\left(0,\mathbf{G}{\upsigma }_{{\text{a}}}^{2}\right)$ and ${\varvec{\uprho}}\sim {\text{N}}(0,\mathbf{I}{\upsigma }_{{\text{pe}}}^{2})$, respectively, where $\mathbf{G}$ is the genomic relationship matrix built according to VanRaden’s Method 1 [27]; ${\phi }_{r}({t}_{ij})$ is the covariate of the regression function with time using LEG or BS; ${t}_{ij}$ is the $j$th time point at which T_V_ was measured throughout the day (24 h) for animal $i$; and $R$ is the order of the LEG or BS for the fixed regression effects, genetic additive random effects, and permanent environmental effects. The same polynomial order was fitted for the additive genetic and permanent environmental effects, as previously suggested [28]. The random residual effect (${e}_{ijklmnr}$) was assumed to be distributed homogeneous or heterogeneous throughout the day. For the latter, residual variance (RV) was modelled considering six periods of 4 h (00h00–04h00, 04h00–08h00, 08h00–12h00, 12h00–16h00, 16h00–20h00, 20h00–24h00)].

The LEG were obtained as proposed by Kirkpatrick et al. [29] and the first three polynomial orders were evaluated. B-spline functions [20] with equally-spaced knots (n = 5, 6, or 7) and degrees (linear—L, quadratic—Q, cubic—C) for the additive genetic and permanent environmental effects were evaluated. The RRM models using BS are referred to as ‘BS*X K*_*a*_* K*_*pe*_’, where *X* = L, Q, or C, that is, the degree of each polynomial segment, and *K*_*a*_ and *K*_*pe*_ are the numbers of random regression coefficients for the additive genetic and permanent environmental effects, respectively. The RRM models using LEG are referred to as ‘LEG *X*’, where *X* is the Legendre orthogonal polynomial order for the additive genetic and permanent environmental effects. Thus, model BSQ88 consists of a quadratic B-spline function with eight random regression coefficients for the additive genetic and permanent environmental effects, and model LEG4 fits additive genetic and permanent environmental regressions based on fourth-order Legendre orthogonal polynomials. The (co)variances components and genetic parameters for T_V_ over 24 h were estimated based on the best model (lowest Bayesian information criterion—BIC and highest accuracy of genomic predictions) using the AIREML algorithm implemented in the BLUPF90 + program [26].

### Statistical models to estimate genetic parameters across environmental gradients

A reaction norm model (RNM) was implemented using the BLUPF90 + program [26] to evaluate GxE interactions, with EG derived by standardizing the dew point as: standardized dew point = (actual dew point – mean dew point)/standard deviation of dew point. The mean and standard deviation of dew point was 23.052 °C and 2.335 °C, respectively. The statistical model used can be described as:

$${y}_{ijklmno}=\alpha 1+{Par}_{k}+{Loc}_{l}+{Hour}_{m}+\beta {\varphi }_{1k}$$

$$+{a}_{0i}1+{a}_{1i}{\varphi }_{1k}+{p}_{0i}1+{p}_{1i}{\varphi }_{1k}+{e}_{ijklmno},$$

where ${y}_{ijklmno}$ is the $o$th observation for T_V_ for animal $i$; $\alpha$ is the intercept; ${Par}_{k}$ is the fixed effect of the $k$th parity of animal $i$; ${Loc}_{l}$ is the fixed effect of the $l$th location (e.g., barn and room within barn) of animal $i$; ${Hour}_{m}$ is the fixed effect of the $m$th measurement hour for animal $i$. $\beta$ is the fixed regression coefficient on EG, ${\varphi }_{1k}$ is the EG vector value $k$, ${a}_{0l}$ and ${a}_{1l}$ are the random regression coefficients for the intercept and slope of the additive genetic effect of individual $i$, ${p}_{0i}$ and ${p}_{1i}$ are the random regression coefficients for the intercept and slope of the random permanent environmental effect of individual $i$, and ${{\text{e}}}_{{\text{ijklmno}}}$ the residual effect, which was assumed distributed homogeneous or heterogeneous throughout the HS period, as described above. The following assumptions were made for the additive genetic effects: $$\left[\begin{array}{c}{{\text{a}}}_{0}\\ {{\text{a}}}_{1}\end{array}\right]\sim {\text{N}}\left(0, \mathbf{G}\otimes {\mathbf{K}}_{\mathbf{a}\mathbf{b}}\right)$$ and $$\left[\begin{array}{c}{{\text{p}}}_{0}\\ {{\text{p}}}_{1}\end{array}\right]\sim {\text{N}}\left(0, \mathbf{I}\otimes {\mathbf{K}}_{\mathbf{c}\mathbf{d}}\right)$$, and ${\mathbf{K}}_{\mathbf{a}\mathbf{b}}$ and ${\mathbf{K}}_{\mathbf{c}\mathbf{d}}$ are both 2 × 2 (co)variance matrices for the intercept and slope effects, with $${\mathbf{K}}_{\mathbf{a}\mathbf{b}}=\left[\begin{array}{cc}{\upsigma }_{0}^{2}& {\upsigma }_{01}\\ {\upsigma }_{10}& {\upsigma }_{1}^{2}\end{array}\right]$$, where ${\upsigma }_{0}^{2}$ is the additive genetic variance for the intercept term, ${\upsigma }_{1}^{2}$ is the additive genetic variance for the slope term, ${\upsigma }_{10}$ and ${\upsigma }_{01}$ are the covariances between the two aforementioned effects, and $${\mathbf{K}}_{\mathbf{c}\mathbf{d}}=\left[\begin{array}{cc}{\upsigma }_{{\text{p}}0}^{2}& {\upsigma }_{{\text{p}}0{\text{p}}1}\\ {\upsigma }_{{\text{p}}1{\text{p}}0}& {\upsigma }_{{\text{p}}1}^{2}\end{array}\right]$$, where ${\upsigma }_{{\text{p}}0}^{2}$ is the permanent environmental variance for the intercept term, ${\upsigma }_{{\text{p}}1}^{2}$ is the permanent environmental variance for the slope term, ${\upsigma }_{{\text{p}}1{\text{p}}0}$ and ${\upsigma }_{{\text{p}}0{\text{p}}1}$ are the covariances between the two aforementioned effects.

Genetic and environmental (co)variance matrices for each time point were defined and calculated as in Oliveira et al. [19]. A one-tailed t-test was performed to determine if there was significant GxE interactions, indicated by the variance of the RNM slope significantly differing from zero. After obtaining (co)variance components, estimates of heritability and repeatability for T_V_ over time were calculated based on the following equations:

$${h}_{i}^{2}=\frac{{\widehat{\sigma }}_{{a}_{i}}^{2}}{{\widehat{\sigma }}_{{a}_{i}}^{2}+{\widehat{\sigma }}_{{pe}_{i}}^{2}+{\widehat{\sigma }}_{{e}_{i}}^{2}},$$

$${r}_{i}=\frac{{\widehat{\sigma }}_{{a}_{i}}^{2}+{\widehat{\sigma }}_{{pe}_{i}}^{2}}{{\widehat{\sigma }}_{{a}_{i}}^{2}+{\widehat{\sigma }}_{{pe}_{i}}^{2}+{\widehat{\sigma }}_{{e}_{i}}^{2}},$$

where ${h}_{i}^{2}$, ${r}_{i}$, ${\widehat{\sigma }}_{{a}_{i}}^{2}$, ${\widehat{\sigma }}_{{pe}_{i}}^{2}$, ${\widehat{\sigma }}_{{e}_{i}}^{2}$ are the estimates of heritability, repeatability, additive genetic variance (VAG), permanent environmental variance (VPE), and RV for the $i$th time point. The estimate of heritability for EG_i_ was calculated as follows [31], ${{\text{h}}}_{{\text{i}}}^{2}=\frac{{\widehat{\upsigma }}_{{{\text{u}}}_{{\text{i}}}}^{2}}{\sum {\widehat{\upsigma }}_{{{\text{n}}}_{{\text{i}}}}^{2}+{\widehat{\upsigma }}_{{\text{e}}}^{2}}$, where ${\widehat{\upsigma }}_{{{\text{u}}}_{{\text{i}}}}^{2}$ is the estimate of the additive genetic variance, which was computed as ${\widehat{\upsigma }}_{{{\text{u}}}_{{\text{i}}}}^{2}={\widehat{\upsigma }}_{{{\text{a}}}_{0}}^{2}+2{\widehat{\upsigma }}_{{{\text{a}}}_{0}{{\text{a}}}_{1}}{\widehat{\uptheta }}_{{\text{i}}}+{\widehat{\upsigma }}_{{{\text{a}}}_{1}}^{2}{({\widehat{\uptheta }}_{{\text{i}}})}^{2}$, and the denominator is the estimate of the phenotypic variance, with $\sum {\widehat{\upsigma }}_{{{\text{n}}}_{{\text{i}}}}^{2}=\sum {\widehat{\upsigma }}_{{{\text{n}}}_{0}}^{2}+2{\widehat{\upsigma }}_{{{\text{n}}}_{0}{{\text{n}}}_{1}}{\widehat{\uptheta }}_{{\text{i}}}+{\widehat{\upsigma }}_{{{\text{n}}}_{1}}^{2}{({\widehat{\uptheta }}_{{\text{i}}})}^{2}$, where ${\text{n}}$ refers to the random effects fitted for T_V_. For the model fitted with heterogenous residual variances, the component ${\widehat{\upsigma }}_{{\text{e}}}^{2}$ was calculated as ${\widehat{\upsigma }}_{{{\text{e}}}_{{\text{i}}}}^{2}={\text{exp}}({{\text{d}}}_{0}+{{\text{d}}}_{1}{\widehat{\uptheta }}_{{\text{i}}})$. The estimate of the genetic correlation between EG_i_ and ${{\text{i}}}^{\mathrm{^{\prime}}}({{\text{r}}}_{{{\text{ii}}}^{\mathrm{^{\prime}}}})$ was calculated as: ${{\text{r}}}_{{{\text{ii}}}^{\mathrm{^{\prime}}}}=\frac{{\widehat{\upsigma }}_{{{\text{u}}}_{{{\text{ii}}}^{\mathrm{^{\prime}}}}}}{\sqrt{{\widehat{\upsigma }}_{{{\text{u}}}_{{\text{i}}}}^{2}{\widehat{\upsigma }}_{{{\text{u}}}_{{{\text{i}}}^{\mathrm{^{\prime}}}}}^{2}}}$, where ${\widehat{\upsigma }}_{{{\text{u}}}_{{{\text{ii}}}^{\mathrm{^{\prime}}}}}$ is the estimate of the covariance of additive genetic effects between EG_i_ and ${{\text{i}}}^{\mathrm{^{\prime}}}$, which was computed as ${\widehat{\upsigma }}_{{{\text{u}}}_{{{\text{ii}}}^{\mathrm{^{\prime}}}}}={\widehat{\upsigma }}_{{{\text{a}}}_{0}}^{2}+{\widehat{\upsigma }}_{{{\text{a}}}_{0}{{\text{a}}}_{1}}{\widehat{\uptheta }}_{{\text{i}}}+{\widehat{\upsigma }}_{{{\text{a}}}_{0}{{\text{a}}}_{1}}{\widehat{\uptheta }}_{{{\text{i}}}^{\mathrm{^{\prime}}}}+{\widehat{\upsigma }}_{{{\text{a}}}_{1}}^{2}{\widehat{\uptheta }}_{{\text{i}}}{\widehat{\uptheta }}_{{{\text{i}}}^{\mathrm{^{\prime}}}}$.

### Comparison of models

The BIC [32] was used to choose the best models, with models with a lower BIC value providing a better fit of the data. The BIC for a model with k estimated parameters and n observations was calculated as:

$${\text{BIC}}=-2{\text{logL}}+{\text{ln}}\left({\text{n}}\right){\text{k}},$$

where $-2{\text{logL}}$ is the restricted maximum log likelihood value of the model. In addition, fivefold cross-validation was used to compare prediction accuracies of the RRM using LEG and BS functions (LEG4 and BSQ88) [33]. For this purpose, the population was randomly allocated to five groups. For a given fold, one group was in turn assigned with missing phenotypic values and used as a validation dataset, and the other four groups were used as the training dataset. Genomic estimated breeding values (GEBV) for the full dataset were calculated for all animals considering the phenotypes from all sows in all groups. The Pearson correlations between GEBV for each time point for sows from the validation dataset and the corresponding GEBV for sows from the full dataset were calculated. The obtained average prediction accuracies across time points were used to select the best model for further analyses.

### Genome-wide association studies

Genome-wide association studies were implemented with a sliding window of 10 consecutive SNPs to map genomic regions that are associated with T_V_, using the postGSf90 software [26]. The same models used to estimate the variance components were implemented for the GWAS. Four HS time-periods throughout the day were used to investigate the genomic regions that are associated with T_V_ at each of these time-periods: (a) from 23h00h to 06h30, which represents the period when T_V_ starts to decrease together with T_a_ and with relatively more thermally comfortable conditions; (b) from 06h30 to 09h30, which represents the period when T_V_ is maintained at thermoneutral levels because the environment is not too hot for the sows; (c) from 09h30 to 18h30, which represents the period when T_V_ increases with increasing T_a_; and (d) from 18h30 to 23h00,which represents the period T_V_ is maintained at a relatively high level because the environment is usually above thermoneutral conditions. Furthermore, three classes of EG were defined to investigate the genomic regions that are associated with T_V_ under different EG conditions. The following three EG classes were defined based on standardized Dew Point units: no to mild HS: − 3.5 to 1.5; moderate HS: − 1.5 to 0.5; and severe HS: 0.5 to 2.5. The GEBV for Tv for an animal was obtained by summing its GEBV at different time points or for different EG classes. For instance, the GEBV for animal ${\text{i}}$ for the four HS time-periods, ${{\text{GEBV}}1}_{{\text{i}}}$, ${{\text{GEBV}}2}_{{\text{i}}}$, ${{\text{GEBV}}3}_{{\text{i}}}$, ${{\text{GEBV}}4}_{{\text{i}}},$ was computed by summing the GEBV at each time point (10-min interval) from 23h00 to 06h30, 06h30 to 09h30, 09h30 to 18h30, 18h30 to 23h00, respectively, as: ${{\text{GEBV}}1}_{{\text{i}}}={{\text{GEBV}}}_{{\text{i}}2305}+{{\text{GEBV}}}_{{\text{i}}2315}+\dots +{{\text{GEBV}}}_{{\text{i}}0635},$

$${{\text{GEBV}}2}_{{\text{i}}}={{\text{GEBV}}}_{{\text{i}}0645}+{{\text{GEBV}}}_{{\text{i}}0655}+\dots +{{\text{GEBV}}}_{{\text{i}}0935},$$

$${{\text{GEBV}}3}_{{\text{i}}}={{\text{GEBV}}}_{{\text{i}}0945}+{{\text{GEBV}}}_{{\text{i}}0955}+\dots +{{\text{GEBV}}}_{{\text{i}}1835},$$

$${{\text{GEBV}}4}_{{\text{i}}}={{\text{GEBV}}}_{{\text{i}}1845}+{{\text{GEBV}}}_{{\text{i}}1855}+\dots +{{\text{GEBV}}}_{{\text{i}}2255}.$$

The SNP effects for each time-period were calculated for T_V_ using the postGSf90 program [26] as:

$${\widehat {\bf{u}}_{\bf{m}}} = {\bf{D}}{{\bf{Z}}^\prime }{[{\bf{ZD}}{{\bf{Z}}^\prime }]^{ - 1}}{\bf{GEB}}{{\bf{V}}_m},$$

where ${\widehat{\mathbf{u}}}_{\mathbf{m}}$ is the vector of the estimated SNP effects for $m$th HS time-period; $\mathbf{D}$ is a diagonal matrix of the weights for the variances of SNP effects ($\mathbf{D}=\mathbf{I}$ for GBLUP), $\mathbf{Z}$ is a matrix relating the genotype (gene content) at each SNP to the individual, and ${\mathbf{G}\mathbf{E}\mathbf{B}\mathbf{V}}_{{\varvec{m}}}$ is the vector of the estimated GEBV for $m$th HS time-period, which includes the GEBV for all animals. The genetic variance explained by SNP $k$ (${\widehat{\sigma }}_{u,k}^{2}$) was estimated as:

$${\widehat{\sigma }}_{u,k}^{2}={\widehat{u}}_{k}^{2}2{p}_{k}\left(1-{p}_{k}\right),$$

where ${p}_{k}$ is the observed allele frequency for the first allele of SNP $k$, and ${\widehat{u}}_{k}^{2}$ is the square of the estimated effect of SNP $k$. The percentage of the total additive genetic variance explained by the $i$th 10-SNP moving window was calculated as:

$$\frac{{{\text{Var}}(\widehat {{a_i}})}}{{\widehat {\sigma _a^2}}} \times 100\% = \frac{{{\text{Var}}\left( {\sum _{{\text{j}} = 1}^{10}{{\bf{z}}_{\text{j}}}{{\widehat {\text{u}}}_{\text{j}}}} \right)}}{{\widehat {\sigma _a^2}}} \times 100\% ,$$

where $\widehat{{a}_{i}}$ is the genetic variance explained by the ${\text{i}}$th window that consists of 10 consecutive SNPs; $\widehat{{\sigma }_{a}^{2}}$ is the total additive genetic variance; ${\mathbf{z}}_{\mathbf{j}}$ is the gene content vector of the ${\text{j}}$th SNP for all individuals, and ${\widehat{{\text{u}}}}_{{\text{j}}}$ is marker effect of the ${\text{j}}$th SNP within the ${\text{i}}$th region. The same method was used to obtain the genomic window variance for the EG classes. Genomic windows that explained more than 0.25% of the genetic variance were considered as relevant genomic regions [34]. To avoid double-counting of SNP effects, only non-overlapping genomic windows were kept for further analyses, as suggested by Fragomeni et al. [35].

### Functional genomic analyses

The genes within the relevant genomic regions were annotated based on the latest pig reference genome Sscrofa 11.1 assembly (http://useast.ensembl.org/Sus_scrofa/Info/Index). Gene Ontology (GO) [36] and Kyoto Encyclopedia of Genes and Genomes (KEGG) [37] enrichment analyses for candidate genes were carried out via the “clusterProfiler” package of R with a cutoff *P*-value < 0.05 and a false discovery rate (FDR) < 0.20 [38].

## Results

**Fig. 1:**
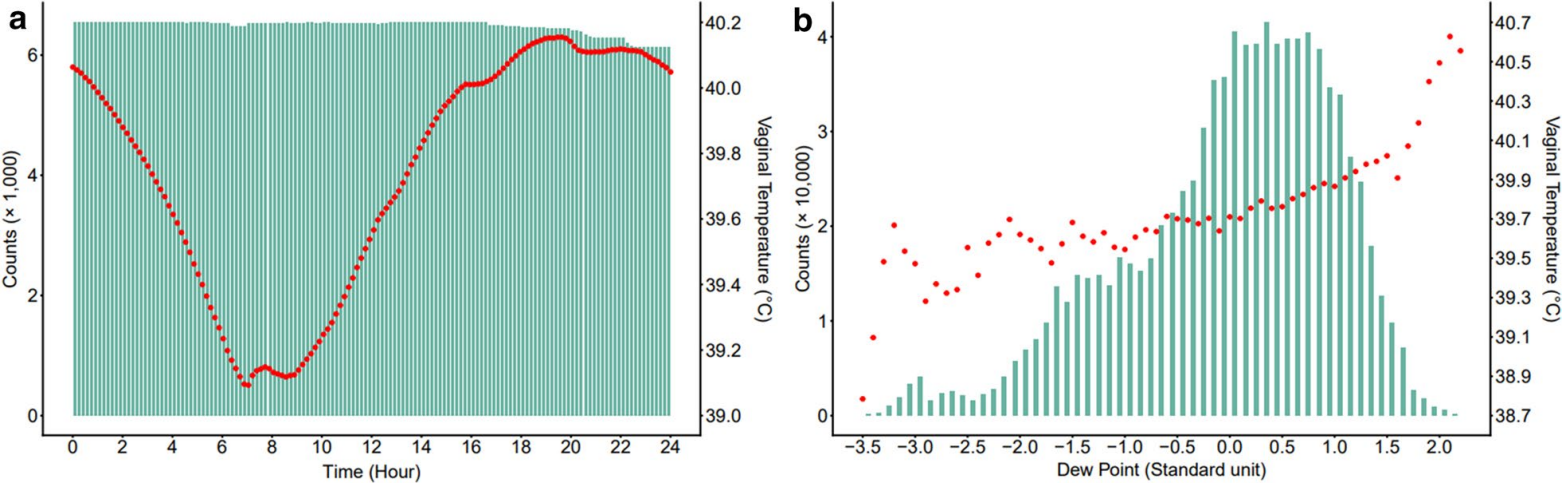
Number of records and average vaginal temperature throughout the day and along environmental gradients. **a** Number of records (green bars) and average vaginal temperature (T_V_) (red dots) for time points (every 10 min) throughout the day and **b** number of records (green bars) and average T_V_ (red dots) along environmental gradients based on dew point values

The number of records and the average Tv for each time point and each EG are presented in Fig. 1. Each time point included more than 6000 records. The T_V_ had a circadian rhythm and remained relatively constant from 06h30 to 09h00 and from 18h30 to 23h00. The number of records was lower for the extreme EG. The average T_V_ showed a distinct pattern along the EG scale, which is based on standardized dew point values. Initially, T_V_ increased significantly at low dew point values. This increase slowed down as the EG value approached 1.7. Beyond this point, the T_V_’s increase became sharp once again (Fig. 1b).

### Statistical model comparisons

Table 1 summarizes the results of the model comparisons based on the logarithm of the likelihood (Log L) and BIC. The number of parameters ranged from 21 to 111 across the models evaluated. The largest number of knots that converged when fitting quadratic BS was 7. For the BS models, BIC decreased as the number of knots and order of the function increased. The BSQ88 model outperformed (lowest BIC) all other models (Table 1). The patterns of the accuracy of genomic prediction over time are shown in Additional file 2: Fig. S2 and the accuracy for T_V_ were 0.72 and 0.13 with models LEG4 and BSQ88, respectively (see Additional file 2: Fig. S2). These results indicate that BSQ88 might have overfitted the data. Thus, LEG4 was used for further analyses.

**Table 1:** Number of model parameters (N), knot position, log likelihood (Log L), Bayesian information criterion (BIC) for random regression models fitted to longitudinal Tv data using B-splines functions or Legendre orthogonal polynomials

Model^a^	N	Knot position^b^	Log L	BIC
B-spline (Linear)				
BSL55	31	5, 363, 721, 1079, 1435	− 419,827	840,079
BSL66	43	5, 291, 577, 863, 1149, 1435	− 397,465	795,520
BSL77	57	5, 243, 481, 719, 957, 1195, 1435	− 387,717	776,218
B-spline (Quadratic)				
BSQ66	43	5, 363, 721, 1079, 1435	− 392,435	785,460
BSQ77	57	5, 291, 577, 863, 1149, 1435	− 386,227	773,237
*BSQ88*	73	5, 243, 481, 719, 957, 1195, 1435	− 384,591	770,186
B-spline (Cubic)				
BSC77	57	5, 363, 721, 1079, 1435	− 389,409	779,602
BSC88	73	5, 291, 577, 863, 1149, 1435	− 385,532	772,006
BSC99	91	5, 243, 481, 719, 957, 1195, 1435	NC	NC
Legendre orthogonal polynomials^c^				
LEG2	7		− 581,803	1,163,702
LEG3	13		− 440,933	882,045
LEG4	21		− 430,771	861,831

NC: the analyses did not converge

^a^in italics is indicated the best model based on BIC

^b^time points of the day (in minutes) where the knots were placed

^c^Legendre orthogonal polynomial of order X (with X = 2, 3, or 4) for the additive genetic and permanent environmental effects

### Variance components and genetic parameters

**Fig. 2:**
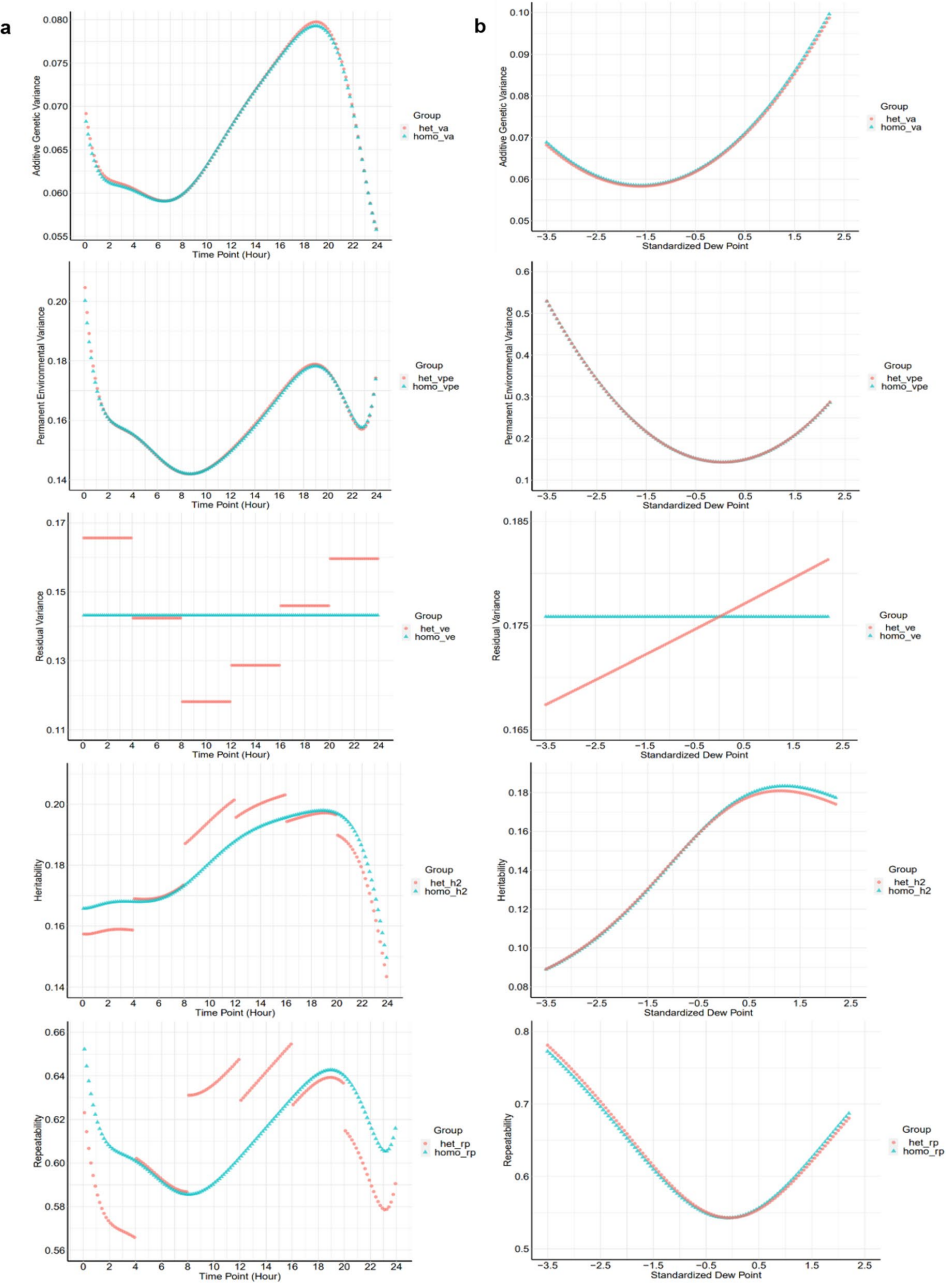
(**a** left column) Additive genetic variance, permanent environmental variance, residual variance, heritability, and repeatability along the time trajectory throughout the day and (**b** right column) continuous environmental gradient for vaginal temperature of lactating sows based on random regression model analyses. Red dot points (●) represent models considering heterogeneous residual variance and green triangles (▲) represent models considering homogeneous residual variance

Figure 2 shows estimates of variance components, heritability, and repeatability for T_V_ throughout the day and along the EG when fitting heterogeneous and homogeneous RV for lactating sows under HS conditions. The model with heterogeneous RV had lower BIC values than those considering homogeneous RV, which implied better goodness of fit. Estimates of VAG, VPE, heritability, and repeatability for T_V_ obtained by the models that fit heterogeneous versus homogeneous RV had similar trends and values. Estimates of heritability (from 0.14 to 0.20) and repeatability (from 0.57 to 0.66) for T_V_ fluctuated over time, with slightly different trends. Heritability estimates for T_V_ increased from 0.09 along the EG scale, with the highest estimate (0.18) at a standardized dew point of 1.2 (25℃), after which it stabilized. Repeatability estimates of T_V_ ranged from 0.54 to 0.77 along the EG.

**Fig. 3:**
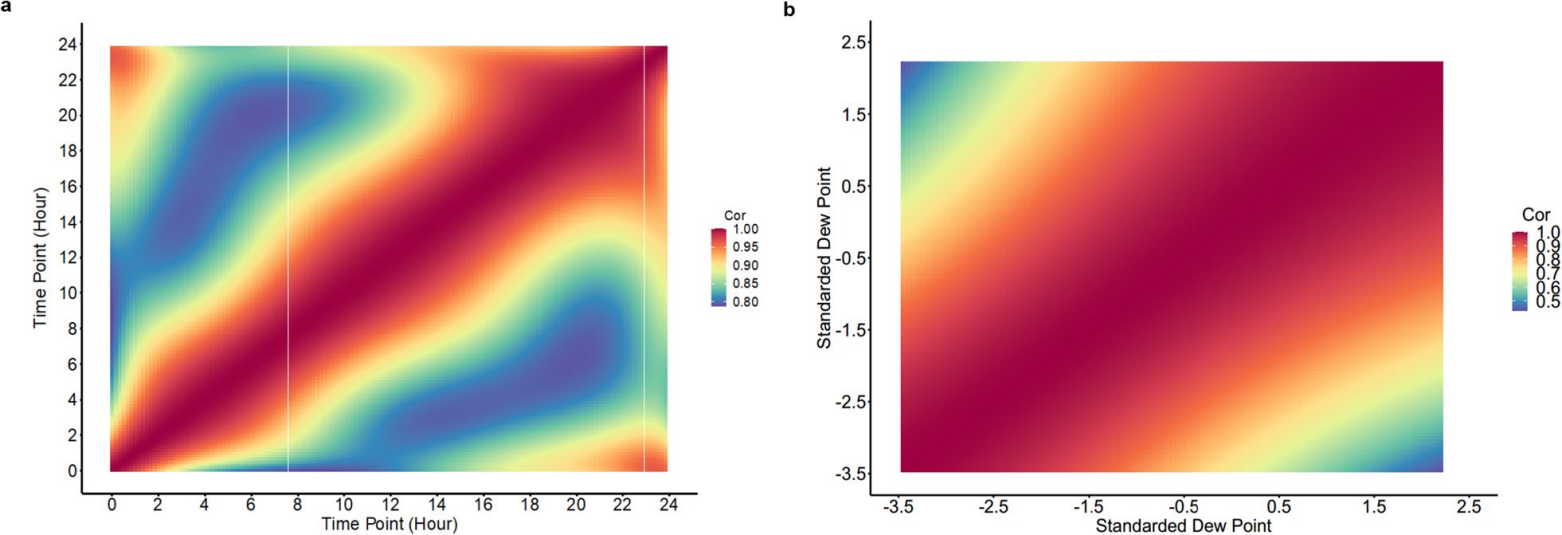
Estimates of genetic correlations (Cor) for vaginal temperature in lactating sows under heat stress between **a** pairs of time points (each 10 min) throughout the day (range: 0.76–1) and **b** pairs of environmental gradients (standardized dew point) (range: 0.44–1)

The trajectories of the estimates of genetic correlations for T_V_ over time and along the EG are shown in Fig. 3a and b, respectively. Over time, the genetic correlation estimates ranged from 0.77 to 1.00, and were lowest for Tv between 09h00–12h00 and 01h00–04h00, and between 04h00–08h00 and 18h00–24h00. For the EG, genetic correlation estimates were positive and moderate to high, ranging from 0.45 to 1.00 (mean correlation: 0.75 ± 0.31) with the lowest estimate observed between the most divergent EG values. The variance of the slope for Tv (0.017) in the RNM model was significantly different from zero using a one-tailed t-test (*P* < 0.05). The estimate of the genetic correlation between the intercept and the slope for T_V_ was 0.33.

### Association analyses

As mentioned above, LEG4 was used for the association analyses. In total, 12, 13, 16, and 10 relevant 10-SNP windows across more than 10 *Sus scrofa* chromosomes (SSC) were identified for T_V_ across the four time-periods 1, 2, 3, and 4, respectively (Table 2). The total additive genetic variance explained by all relevant genomic windows for the four HS time-periods was 16.9%. Based on the candidate genes identified, four and one KEGG pathways were significantly enriched for the HS time-periods 3 and 4, respectively (see Additional file 3: Tables S1 and S2). For the GO analyses, 12, 21, and 13 terms were significantly enriched for HS time-periods 1, 3 and 4, respectively (see Additional file 3: Table S3). The results from the KEGG and GO analyses indicate that development (embryonic skeletal system development), neural system (glutamatergic synapse), and cardiac disease related pathways (hypertrophic cardiomyopathy, dilated cardiomyopathy) were the most enriched pathways based on the candidate genes identified.

**Table 2:** Relevant genomic regions identified for the four heat stress (HS) time-periods

Time-period^a^	Chromosome bp positions	% of variance explained	Positional candidate genes
HS time-period 1	10:59,369,633:59,776,918	0.66	*CAMK1D, RNU6ATAC39P, CDC123, NUDT5, SEC61A2, KATNAL1, USPL1, ALOX5AP, EFTUD2, HIGD1B, GJC1, ADAM11, DBF4B, CCDC43, MEIOC, FZD2, GPATCH8, ITGA2B, FAM171A2, GRN, SLC25A39, RUNDC3A, SLC4A1, HOXB4, MIR10A, HOXB5, HOXB6, HOXB7, HOXB8, HOXB9, MIR196A1, HOXB13, TTLL6, CALCOCO2, ATP5MC1, UBE2Z, SNF8, GIP, IGF2BP1, PISD, PRR14L, DEPDC5, YWHAH, SLC5A1, SLC5A4, RANBP3L, SLC1A3, U6, MYLK2, FOXS1, DUSP15, TTLL9, PDRG1, XKR7, CCM2L, HCK, TM9SF4, PLAGL2, POFUT1, KIF3B, ASXL1, MIR148A, SDK1, SNORA70*
(T_V_: 39.741 ± 0.724 DP: 22.080 ± 2.060 Total explained variance: 4.086%)	16:21,601,170:21,965,767	0.45	
	12:18,499,751:18,974,836	0.379	
	17:35,507,357:36,063,488	0.349	
	3:3,029,529:3,257,462	0.317	
	18:46,374,852:46,983,072	0.293	
	12:24,812,751:25,420,666	0.284	
	11:7,020,997:7,540,141	0.280	
	17:67,332,252:67,844,392	0.279	
	17:65,356,677:65,763,082	0.275	
	14:48,306,021:48,731,419	0.274	
	6:134,063,309:134,266,656	0.253	
HS time-period 2	16:21,601,170:21,965,767	0.490	*CAMK1D, CDC123, NUDT5, SEC61A2, RNU6ATAC39P, KATNAL1, USPL1, ALOX5AP, PSMB3, PCGF2, CISD3, MLLT6, SRCIN1, ARHGAP23, GPR179, MRPL45, EPOP, ADAM11, GJC1, SLC25A39, RUNDC3A, SLC4A1, DBF4B, MEIOC, FZD2, ITGA2B, FAM171A2, EFTUD2, HIGD1B, U6, GPATCH8, PISD, PRR14L, DEPDC5, YWHAH, SLC5A1, SLC5A4, RANBP3L, SLC1A3, MYLK2, FOXS1, DUSP15, TTLL9, PDRG1, XKR7, CCM2L, HCK, TM9SF4, PLAGL2, POFUT1, KIF3B, ASXL1, MIR148A, SDK1, CEBPG, PEPD, CHST8, KCTD15, CALCR, GNGT1, CDK6, SAMD9, HEPACAM2, VPS50, ssc-mir-489*
(T_V_: 39.134 ± 0.620 DP: 22.844 ± 2.084 Total explained variance: 4.840%)	10:59,369,633:59,776,918	0.481	
	3:3,029,529:3,257,462	0.414	
	14:48,306,021:48,731,419	0.394	
	18:46,374,852:46,983,072	0.383	
	12:18,499,751:18,974,836	0.380	
	6:43,090,860:43,731,895	0.359	
	11:7,020,997:7,540,141	0.318	
	17:65,356,677:65,763,082	0.292	
	17:67,332,252:67,844,392	0.289	
	12:23,350,211:23,791,623	0.279	
	17:35,507,357:36,063,488	0.271	
	16:21,601,170:21,965,767	0.490	
HS time-period 3	16:21,548,342:21,840,246	0.364	*CAMK1D, CDC123, NUDT5, SEC61A2, DHTKD1, UPF2, RNU6ATAC39P, KATNAL1, USPL1, ALOX5AP, PIP4K2B, PSMB3, PCGF2, CISD3, MLLT6, SRCIN1, ARHGAP23, SOCS7, GPR179, MRPL45, EPOP, ADAM11, GJC1, SLC25A39, RUNDC3A, SLC4A1, DBF4B, CCDC43, MEIOC, FZD2, ITGA2B, FAM171A2, GRN, EFTUD2, HIGD1B, U6, GPATCH8, PISD, PRR14L, DEPDC5, YWHAH, SLC5A1, SLC5A4, NADK2, RANBP3L, MIR148A, SDK1, ZFAT, WNT10B, WNT1, CACNB3, DDX23, RND1, CCDC65, FKBP11, DDN, C12orf75, CCNT1, TEX49, ADCY6, PRKAG1, RHEBL1, DHH, ARF3, CEBPG, PEPD, CHST8, KCTD15, GRID2, FSTL5*
(T_V_: 39.771 ± 0.729 DP: 23.843 ± 2.478 Total explained variance: 4.793%)	17:67,332,252:67,844,392	0.353	
	8:144,423,490:145,098,780	0.351	
	18:46,374,852:46,983,072	0.327	
	6:43,097,060:43,829,382	0.319	
	8:125,810,305:126,470,348	0.314	
	3:3,029,529:3,257,462	0.301	
	5:14,539,752:15,115,540	0.294	
	11:7,020,997:7,540,141	0.290	
	8:49,296,088:50,537,893	0.282	
	10:59,507,616:59,997,776	0.282	
	12:18,499,751:18,974,836	0.276	
	14:48,306,021:48,731,419	0.272	
	4:7,002,305:7,146,525	0.265	
	14:119,028,054:119,983,210	0.252	
	12:23,321,216:2,3760,296	0.251	
HS time-period 4	10:59,507,616:59,997,776	0.398	*CAMK1D, RNU6ATAC39P, CDC123, NUDT5, SEC61A2, DHTKD1, UPF2, EFTUD2, HIGD1B, GJC1, ADAM11, DBF4B, CCDC43, MEIOC, U6, FZD2, GPATCH8, ITGA2B, FAM171A2, GRN, SLC25A39, RUNDC3A, SLC4A1, NADK2, RANBP3L, MYLK2, FOXS1, DUSP15, TTLL9, PDRG1, XKR7, CCM2L, HCK, TM9SF4, PLAGL2, POFUT1, KIF3B, ASXL1, NT5C3A, FKBP9, RP9, KBTBD2, AVL9, PDE1C, INCENP, BEST1, RAB3IL1, FADS3, U1, ZFAT, GRID2*
(T_V_: 40.125 ± 0.687 DP: 23.226 ± 1.996 Total explained variance: 3.141%)	3:64,224,248:65,386,969	0.374	
	4:7,002,305:7,146,525	0.335	
	17:67,332,252:67,844,392	0.328	
	17:35,507,357:36,063,488	0.310	
	18:40,471,432:40,882,202	0.304	
	16:21,548,342:21,840,246	0.291	
	2:9,425,931:9,613,380	0.273	
	8:125,810,305:126,470,348	0.265	
	12:18,499,751:18,974,836	0.263	

HS: heat stress

^a^Time-period: mean ± standard deviation of vaginal temperature (Tv), dew point (DP) and total explained variance for each stage; 1: from 23h00 to 06h30, 2: from 06h30 to 09h30, 3: from 09h30 to 18h30, and 4: from 18h30 to 23h00

Eighteen, 15, and 14 10-SNP windows were identified for T_V_ for the three EG classes (mild HS: − 3.5 to 1.5; moderate HS: − 1.5 to 0.5; and severe HS: 0.5 to 2.5, respectively) (Table 3). The total additive genetic variance explained by all the relevant genomic windows for the three EG classes was 16.3%. No significant KEGG pathway and, respectively, 30, 23, and 58 GO terms (see Additional file 3: Table S4) were significantly enriched based on the candidate genes (see Additional file 3: Table S5) identified for the three EG classes. Enriched GO terms were mainly associated with development (e.g., skeletal system development), energy metabolism (e.g., fatty acid biosynthesis processes), and hormone regulation (e.g., regulation of secretion).

**Table 3:** Relevant genomic regions identified for three environmental gradient (EG) classes

Environmental gradient (EG) class^a^	Position	Explained variance (%)	Genes
EG class 1	18:46,374,852:46,983,072	0.656	*CAMK1D, CDC123, NUDT5, SEC61A2, RNU6ATAC39P, IGF2BP1, HOXB1, HOXB2, HOXB3, HOXB4, HOXB5, HOXB6, HOXB7, HOXB9, HOXB13, TTLL6, CALCOCO2, ATP5MC1, UBE2Z, SNF8, GIP, MIR196A1, MIR10A, KCNH8, PISD, PRR14L, DEPDC5, YWHAH, SLC5A1, SLC5A4, U6, CDH10, RANBP3L, MYLK2, FOXS1, DUSP15, TTLL9, PDRG1, XKR7, CCM2L, HCK, TM9SF4, PLAGL2, POFUT1, KIF3B, ASXL1, NT5C3A, FKBP9, RP9, KBTBD2, AVL9, PDE1C, MIR148A, INCENP, BEST1, RAB3IL1, FADS3, ZFAT, BCAR3, FNBP1L*
(T_V_: 39.535 ± 0.734 DP: 18.217 ± 1.176 Total explained variance: 5.688%)	14:142,203,506:142,387,878	0.490	
	16:11,509,437:12,226,132	0.447	
	17:67,332,252:67,844,392	0.414	
	7:127,981,346:128,414,726	0.380	
	2: 9,425,931: 9,613,380	0.379	
	12:24,782,254:25,386,252	0.349	
	4:123,450,469:123,692,404	0.318	
	13: 5,982,176: 6,582,320	0.317	
	17:35,507,357:36,063,488	0.293	
	16:21,577,390:21,860,428	0.284	
	4: 7,002,305: 7,146,525	0.280	
	10:59,377,853:59,815,833	0.279	
	14:48,306,021:48,731,419	0.275	
	18:40,475,973:40,910,375	0.274	
	17:65,356,677:65,763,082	0.253	
EG class 2	10:59,377,853:59,815,833	0.481	*SMOC2, CAMK1D, CDC123, NUDT5, SEC61A2, RNU6ATAC39P, IGF2BP1, HOXB4, HOXB5, HOXB6, HOXB7, HOXB8, HOXB9, HOXB13, TTLL6, CALCOCO2, ATP5MC1, UBE2Z, SNF8, GIP, MIR196A1, MIR10A, PTPN11, RPL6, HECTD4, TRAFD1, NAA25, ERP29, TMEM116, MAPKAPK5, ALDH2, BICDL1, RAB35, GCN1, RPLP0, PXN, U6, MSI1, COX6A1, GATC, SRSF9, DYNLL1, COQ5, RNF10, POP5, MLEC, ACADS, SPPL3, HNF1A, C12orf43, OASL, C12orf76, ANKRD13A, GIT2, GLTP, SIRT4, PLA2G1B, PISD, PRR14L, DEPDC5, YWHAH, SLC5A1, SLC5A4, U4, CABP1, UNC119B, CDH10, RANBP3L, NT5C3A, FKBP9, RP9, KBTBD2, AVL9, PDE1C, MIR148A, INCENP, BEST1, RAB3IL1, FADS3, SDK1, CEBPG, PEPD, CHST8, KCTD15, GRID2, FSTL5*
(T_V_: 39.687 ± 0.743DP: 22.403 ± 1.283Total explained variance: 5.476%)	18:46,374,852:46,983,072	0.430	
	14:142,174,006:142,343,216	0.360	
	16:21,577,390:21,860,428	0.342	
	12:24,812,751:25,420,666	0.326	
	17:65,356,677:65,763,082	0.320	
	8:125,810,305:126,470,348	0.300	
	17:67,332,252:67,844,392	0.291	
	2: 9,425,931: 9,613,380	0.282	
	16:11,509,437:12,226,132	0.276	
	6:43,097,060:43,829,382	0.269	
	18:40,471,432:40,882,202	0.266	
	7:127,981,346:128,414,726	0.264	
	14:48,306,021:48,731,419	0.259	
	1: 1,049,272: 1,334,349	0.256	
	3: 3,029,529: 3,257,462	0.252	
	14:39,171,016:41,112,851	0.252	
	8:49,296,088:50,537,893	0.250	
EG class 3	10:59,377,853:59,815,833	0.402	*CAMK1D, CDC123, NUDT5, SEC61A2, RNU6ATAC39P, KATNAL1, USPL1, ALOX5AP, IGF2BP1, HOXB4, HOXB5, HOXB6, HOXB7, HOXB8, HOXB9, HOXB13, TTLL6, CALCOCO2, ATP5MC1, UBE2Z, SNF8, GIP, MIR196A1, MIR10A, PTPN11, RPL6, HECTD4, TRAFD1, NAA25, ERP29, TMEM116, MAPKAPK5, ALDH2, BICDL1, RAB35, GCN1, RPLP0, PXN, U6, MSI1, COX6A1, GATC, SRSF9, DYNLL1, COQ5, RNF10, POP5, MLEC, ACADS, SPPL3, HNF1A, C12orf43, OASL, C12orf76, ANKRD13A, GIT2, GLTP, SIRT4, PLA2G1B, U4, CABP1, UNC119B, CDH10, NADK2, RANBP3L, MIR148A, INCENP, BEST1, RAB3IL1, FADS3, SDK1, UTS2, TNFRSF9, CAMTA1, PARK7, ERRFI1, CEBPG, PEPD, CHST8, KCTD15, GRID2, FSTL5*
(T_V_: 39.874 ± 0.765 DP: 25.310 ± 0.743 Total explained variance: 5.101%)	18:46,374,852:46,983,072	0.395	
	14:142,174,006:142,343,216	0.339	
	8:125,810,305:126,470,348	0.322	
	8:49,296,088:50,537,893	0.321	
	17:65,356,677:65,763,082	0.321	
	16:21,548,342:21,840,246	0.308	
	6:43,097,060:43,829,382	0.297	
	17:67,385,528:67,932,872	0.288	
	2: 9,425,931: 9,613,380	0.278	
	11:7,020,997:7,540,141	0.275	
	3: 3,029,529: 3,257,462	0.274	
	16:11,509,437:12,226,132	0.268	
	12:24,812,751:25,420,666	0.254	
	8:144,423,490:145,098,780	0.254	
	6:68,449,792:68,823,162	0.253	
	14:39,171,016:41,112,851	0.252	

^a^Environmental gradient: mean ± standard deviation of vaginal temperature (Tv), dew point (DP), and total explained variance for each environmental gradient (standardized Dew point) class (no to mild heat stress: − 3.5 to 1.5; moderate heat stress: − 1.5 to 0.5; severe heat stress: 0.5–2.5)

**Fig. 4:**
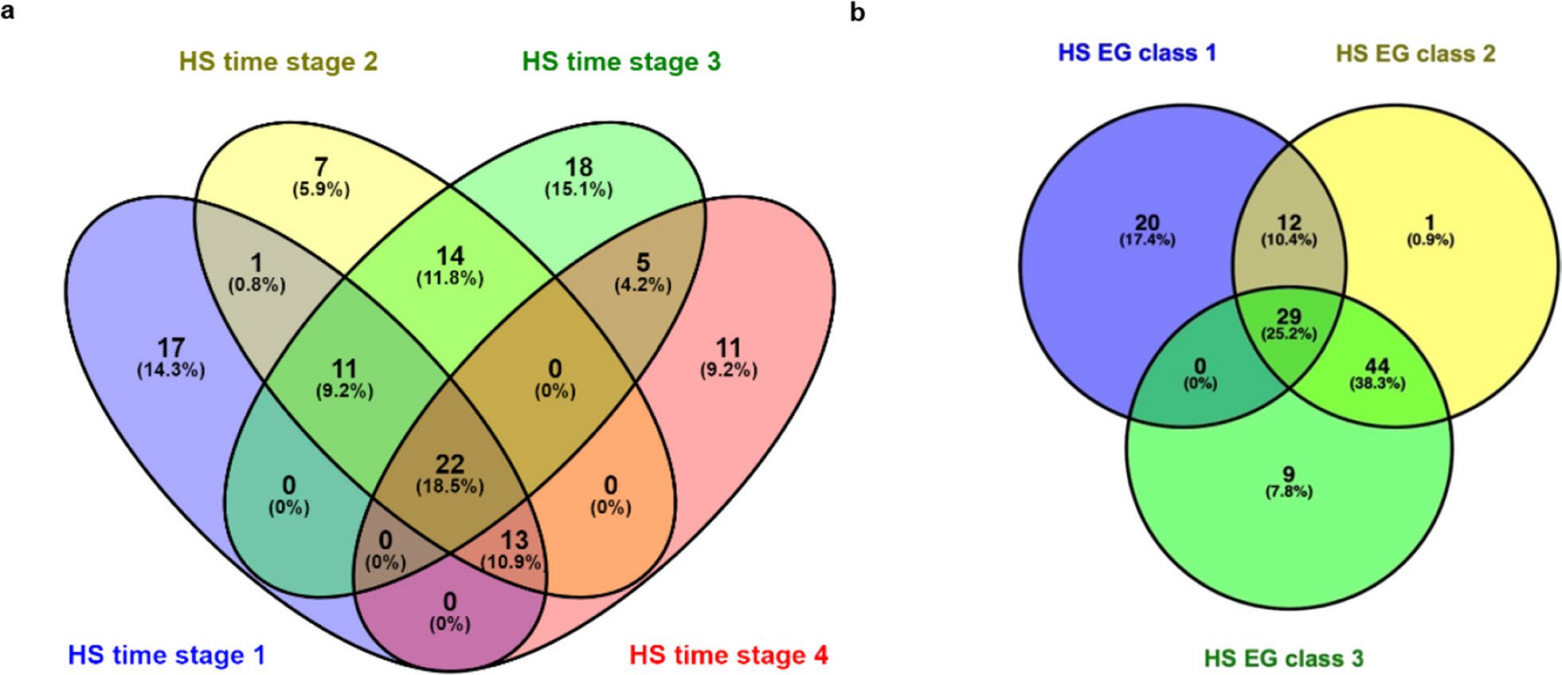
Venn diagram of the count of candidate genes identified based on **a** heat stress (HS) time-periods and **b** environmental gradient class. The four potential HS time-periods throughout the day were defined as (1) from 23h00 to 06h30, representing when vaginal temperature (T_V_) starts to decrease with decreased ambient temperature (T_a_) and with relatively more comfortable environmental conditions; (2) from 06h30 to 09h30, representing T_V_ maintained at a relatively low level when environment is not too hot for the sows; (3) from 09h30 to 18h30, representing the time in which T_V_ starts to increase with increasing T_a_; (4) from 18h30 to 23h00, representing T_V_ maintained at a relatively high level when T_a_ is usually above thermoneutral conditions. Each two standardized Dew point units was considered as an environmental gradient class (no to mild HS: − 3.5 to 1.5; moderate HS: − 1.5 to 0.5; severe HS: 0.5–2.5)

Two important genomic regions were identified to be associated with Tv across all HS time-periods and all EG classes, on SSC10 (59.370–59.998 Mb) and SSC16 (21.548–21.966 Mb). Candidate genes within these regions are involved in immunity (*CDC123* and *CAMK1d)*, protein transport (*SEC61A2*), and energy metabolism (*NUDT5*) functions. The overlapping and unique candidate genes regulating T_V_ across the four HS time-periods and the three EG classes are presented in a Venn diagram in Fig. 4. HS time-stage 3 had the most uniquely enriched genes (18), while HS time-stage 2 had the least number of uniquely enriched genes (7). One, 16, 21, and 7 significant KEGG pathways were detected based on the uniquely enriched genes for the four respective HS time-periods (Table 4). Furthermore, EG classes 1, 2, and 3 had 20, 1, and 9 uniquely enriched genes, respectively. Twenty-nine genes were shared by all three EG classes. Three, five, and three significant KEGG pathways were detected based on the uniquely enriched genes for EG classes 1, 2, and 3, respectively (Table 5).

**Table 4:** Significantly enriched (*P* < 0.05) KEGG pathways using uniquely enriched positional candidate genes for Tv for the four heat stress (HS) time-periods

Time-period^a^	Term	Number of unique genes^b^	Total number of genes^c^	*P*-value	FDR
HS time-period 1	ssc04137: Mitophagy—animal	17	64	0.047	0.195
(T_V_: 39.741 ± 0.724DP: 22.080 ± 2.060)					
HS time-period 2	ssc04744: Phototransduction	7	68	0.006	0.012
(T_V_: 39.134 ± 0.620DP: 22.844 ± 2.084)	ssc04727: GABAergic synapse			0.020	0.012
	ssc05032: Morphine addiction			0.021	0.012
	ssc04713: Circadian entrainment			0.022	0.012
	ssc04724: Glutamatergic synapse			0.025	0.012
	ssc04725: Cholinergic synapse			0.025	0.012
	ssc04726: Serotonergic synapse			0.026	0.012
	ssc04380: Osteoclast differentiation			0.028	0.012
	ssc04728: Dopaminergic synapse			0.029	0.012
	ssc04926: Relaxin signaling pathway			0.029	0.012
	ssc04371: Apelin signaling pathway			0.031	0.012
	ssc04723: Retrograde endocannabinoid signaling			0.033	0.012
	ssc05034: Alcoholism			0.038	0.012
	ssc04062: Chemokine signaling pathway			0.041	0.012
	ssc05167: Kaposi sarcoma-associated herpesvirus infection			0.044	0.012
	ssc05170: Human immunodeficiency virus 1 infection			0.049	0.012
HS time-period 3	ssc04916: Melanogenesis	18	70	< 0.001	0.014
(T_V_: 39.771 ± 0.729 DP: 23.843 ± 2.478)	ssc04921: Oxytocin signaling pathway			0.001	0.016
	ssc04934: Cushing syndrome			0.001	0.016
	ssc04213: Longevity regulating pathway—multiple species			0.002	0.035
	ssc05217: Basal cell carcinoma			0.003	0.035
	ssc04211: Longevity regulating pathway			0.005	0.049
	ssc05410: Hypertrophic cardiomyopathy			0.005	0.049
	ssc05414: Dilated cardiomyopathy			0.006	0.049
	ssc04371: Apelin signaling pathway			0.012	0.067
	ssc04550: Signaling pathways regulating pluripotency of stem cells			0.013	0.067
	ssc04261: Adrenergic signaling in cardiomyocytes			0.013	0.067
	ssc05224: Breast cancer			0.013	0.067
	ssc05226: Gastric cancer			0.014	0.067
	ssc04150: mTOR signaling pathway			0.015	0.067
	ssc04390: Hippo signaling pathway			0.015	0.067
	ssc05225: Hepatocellular carcinoma			0.016	0.068
	ssc04310: Wnt signaling pathway			0.017	0.068
	ssc05207: Chemical carcinogenesis—receptor activation			0.025	0.091
	ssc05205: Proteoglycans in cancer			0.026	0.091
	ssc04714: Thermogenesis			0.032	0.108
	ssc04710: Circadian rhythm			0.042	0.136
HS time-period 4	ssc00240: Pyrimidine metabolism	11	51	0.018	0.006
(T_V_: 40.125 ± 0.687 DP: 23.226 ± 1.996)	ssc04924: Renin secretion			0.023	0.006
	ssc04742: Taste transduction			0.024	0.006
	ssc01232: Nucleotide metabolism			0.027	0.006
	ssc05032: Morphine addiction			0.031	0.006
	ssc03040: Spliceosome			0.042	0.006
	ssc00230: Purine metabolism			0.043	0.006

HS: heat stress; FDR: false discovery rate

^a^Time-period: mean ± standard deviation was showed for each stage: 1: from 23h00 to 06h30, 2: from 06h30 to 09h30, 3: from 09h30 to 18h30, and 4: from 18h30 to 23h00

^b^Number of unique genes: number of genes was uniquely enriched in each HS stage

^c^Total number of genes: number of all genes enriched in each HS stage

**Table 5:** Significantly enriched (*P* < 0.05) KEGG pathways using uniquely enriched candidate genes identified for the three environmental gradient classes

Environmental gradient (EG) class^a^	Term	Number of unique genes^b^	Total number of genes^c^	*P*-value	FDR
EG class 1	ssc00514: Other types of O-glycan biosynthesis	20	61	0.016	0.027
(T_V_: 39.535 ± 0.734 DP: 18.217 ± 1.176)	ssc04666: Fc gamma R-mediated phagocytosis			0.031	0.027
	ssc04550: Signaling pathways regulating pluripotency of stem cells			0.047	0.027
EG class 2	ssc04973: Carbohydrate digestion and absorption	1	86	0.016	0.034
(T_V_: 39.687 ± 0.743 DP: 22.403 ± 1.283)	ssc04978: Mineral absorption			0.018	0.034
	ssc04976: Bile secretion			0.028	0.034
	ssc04114: Oocyte meiosis			0.040	0.034
	ssc04110: Cell cycle			0.041	0.034
EG class 3	ssc00760: Nicotinate and nicotinamide metabolism	9	82	0.028	0.076
(T_V_: 39.874 ± 0.765DP: 25.310 ± 0.743)	ssc04080: Neuroactive ligand-receptor interaction			0.030	0.076
	ssc04730: Long-term depression			0.046	0.076

EG: environmental gradient; FDR: false discovery rate

^a^Stage: mean ± standard deviation of vaginal temperature (Tv) and dew point (DP) for each environmental gradient (standardized dew point) class (no to mild heat stress: − 3.5 to 1.5; moderate heat stress: − 1.5 to 0.5; severe heat stress: 0.5–2.5)

^b^Number of unique genes: number of genes was uniquely enriched in each EG class

^c^Total number of genes: number of all genes enriched in each EG class

## Discussion

Climate change threatens worldwide livestock production and causes substantial economic and animal welfare issues. Understanding the genetic basis of an animal’s HS response, including identification of novel indicator traits such as T_V_, is paramount for designing effective strategies for breeding more resilient and productive pigs. In this study, genetic parameters and GWAS analyses were conducted for T_V_ that was automatically measured every 10 min in Landrace × Large White lactating sows under HS conditions. The datasets of automatically-measured T_V_ and within-barn environmental measurements provided an opportunity to explore the genetic basis of longitudinal variability in Tv during lactation.

### Comparison of models

The order of the BS had a greater impact on estimates of variance components than the number of knots. The genetic parameter estimates followed similar trends for all cubic BS models. Lower BIC values for models with a larger number of parameters have been reported previously (e.g., [20, 39, 40]). Although LEG models had larger BIC values than BS models, the computing times of the LEG models were much shorter. The lower accuracy of genomic prediction obtained with the RRM model using BS indicated that BS models may overfit the data, and based on the cross-validation analyses the LEG models were considered to be better.

### Estimates of genetic parameters and variance components

To our knowledge, this study presents the first estimates of heritability and variance components for T_V_ over time and along EG under HS conditions in lactating sows. The use of longitudinal automatically-measured T_V_ is expected to yield more accurate estimates. Estimates of variance components along the EG scale were generally larger than those across time periods, while the heritability estimates across periods were higher. The moderate level of the average heritability estimate for T_V_ obtained with the RRM with LEG (0.18) indicates that T_V_ can be included in selection indexes as a heat tolerance indicator. Another measure of internal temperature, rectal temperature has been reported to have similar heritability estimates in lactating Holstein cows (0.17 [41] and 0.15 to 0.31 [42]). In those studies, the temperature was collected only once a day, which did not allow changes in heritability estimates and genetic variances throughout the day or with climatic conditions to be evaluated. Nevertheless, further studies are needed to evaluate the genetic correlations between T_V_ and other economically important traits (e.g., weaning weight) in pigs. If T_V_ can only be measured during a short time-window, the time-period from 1200 to 1600 h is recommended because it had the highest average heritability (0.20) and repeatability (0.64).

Trends in heritability estimates across time and across EG were similar to trends in average Tv (Fig. 1). A substantial increase in average T_V_ was observed after the 1.7 value for EG (dew point: 27 Celsius degree), which suggests that animals may experience severe HS above this dew point and may not be able to maintain body temperature homeostasis, thus needing cooling devices to maintain animal welfare. Trends in heritability estimates along the EG indicated that genetics has an increasing role in controlling T_V_ as the dew point increases. Thus, greater selection response to T_V_ is expected in a hotter environment due to the greater genetic variation, likely because more physiological and behavioral processes are involved in body temperature regulation under severe HS conditions [43]. Previous studies reported that heritability estimates for rectal temperature under HS conditions ranged from 0.11 to 0.36 in many species, which is in line with our results [41, 44, 45]. Also, the high VPE and repeatability estimates for Tv at the extremes of the EG scale indicate that relatively constant and accurate records could be obtained under extreme or mild HS conditions (Fig. 2b).

The variance for the slope for T_V_ from the RNM models was significantly different from zero, which indicates that GxE interactions exist when lactating sows are kept under HS conditions. The estimate of the genetic correlation being lowest (0.47) between extreme EG also supports the existence of GxE interactions and implies that animals will be ranked differently along the EG scale (Fig. 3b). Heat resilient individuals that can maintain performance levels under HS contribute to a greater profitability under HS conditions [46]. Cooling devices (i.e., sprinkler systems and evaporative cooling system) and changes in animal behavior (i.e., reduced feed intake, reduced milk production, and laying down for longer times) can also mitigate the negative effects of HS and improve the animals’ actual performance by reducing air temperature [2] or metabolic heat production [47]. Future research on the genetics of longitudinal Tv should consider larger datasets with greater environmental variability and different populations.

### GWAS

Understanding the genetic mechanisms that underlie changes in T_V_ of lactating sows under HS is important to alleviate adverse effects from HS and to select heat stress resilient animals. This is the first study to report GWAS results for continuously-recorded T_V_ in lactating sows under HS conditions, mainly because obtaining relevant data is difficult. In this study, we performed a GWAS to investigate the dynamic regulatory functions of HS-linked genomic regions, genes, and QTL. Automatically-measured body temperature data and environmental records capture more variation and allow a more accurate detection of genomic regions as compared to traditional reaction norm models based on performance traits and data from public weather stations.

Multiple genomic regions on different chromosomes but with relatively small contributions to the total additive genetic variation of T_V_ were identified across the different measurement periods, indicating that T_V_ is a highly polygenic trait (Tables 2 and 3). Previous reports have indicated that rectal temperature, which is strongly genetically correlated with T_V_ in dairy cattle, sows, and sheep (r ≥ 0.8), is also a highly polygenic trait [15, 48–50]. Our results showed that some effects of the identified genomic regions were consistent throughout the entire HS period, while most genes or genomic regions associated with T_V_ regulation are involved in different HS time-periods and EG classes. This suggests that genetic regulation of T_V_ is complex and influenced by both common and unique genomic regions. Regulation of T_V_ can also be affected by farm management (i.e., cooling device), environmental conditions (i.e., good ventilation), and animal physiological state [16, 43].

Six genes were identified for T_V_ under HS conditions: *CAMK1D*, *RNU6ATAC39P*, *CDC123*, *NUDT5*, *SEC61A2*, and *RANBP3L*, many of which have previously been reported to be related to HS. The genes *CAMK1D* and *CDC123* are mainly related to immunity. Heat stress can result in intestinal and systematic inflammation and thus in suppressing the innate immune function and increasing the animals’ susceptibility to diseases [51]. Many studies have also demonstrated that HS has detrimental effects on immunity and negatively affects health in cattle [52, 53], pigs [54, 55], sheep [56, 57], goats [58, 59], and poultry [60, 61]. Another study reported that *SEC61A2* was expressed during HS [62].

### GWAS enrichment over time

The results for KEGG pathway enrichment were similar for the first two HS time-periods, and the enriched pathways were mainly related to the nervous system (i.e., glutamatergic synapse) and platelet activation. The pathways that were enriched for HS time-period 3 were mainly related to signaling (i.e., oxytocin signaling pathway, mTOR signaling pathway, and hippo signaling pathway), cardiac disease (i.e., hypertrophic cardiomyopathy, dilated cardiomyopathy), and hormone synthesis and regulation (i.e., aldosterone synthesis and secretion). Previous studies revealed that the cardiovascular system plays a key role in human thermoregulation because heat dissipation is accompanied by cardiovascular adjustments, including increased cardiac output through a combination of elevations in heart rate and cardiac contractility and elevations in sympathetic activity for reduced blood flow and blood volume [63–65]. The cardiovascular system can be negatively affected by HS [66]. Occurrences of cardiovascular and respiratory mortality have been shown to increase significantly under HS conditions, especially in tropical areas [67, 68]. Under HS conditions, animals can experience severe dehydration, which would activate the renin–angiotensin–aldosterone system to maintain fluid and electrolyte balance [69]. Previous studies in cattle have shown that aldosterone concentration declines substantially under HS [70]. In our study, the pathways that were enriched for the HS time-period 4 were associated with various functions, including signaling (i.e., calcium signaling pathway and oxytocin signaling pathway), protein export, and platelet activation.

Many GO terms that were enriched for the four HS time-periods were related to development (i.e., embryonic skeletal system morphogenesis, embryonic skeletal system development), metabolic process (i.e., glucocorticoid metabolic process, carbohydrate metabolic process), nervous system (i.e., dendrite development, regulation of neuron projection development, and dendritic spine morphogenesis), and immunity (i.e., lymphocyte mediated immunity, natural killer cell mediated immunity, and leukocyte mediated immunity). This suggests that a sow’s response to HS is a complex process that involves a series of nervous, physiological, cellular, and molecular processes that can result in greater stress resilience, which is in line with previous studies [71, 72].

To identify which biological pathways play key and unique roles in response to HS and to better understand their functions, KEGG enrichment analyses of uniquely enriched genes were performed for each HS time-period. Although many similarities were found between the four HS time-periods, specific biologic functions were identified for each period. For HS time-period 1, only one significant pathway was identified, i.e. mitophagy-animal, which is known to increase resistance to diverse stressors and improve longevity [73]. Several studies have reported that mitophagy is impaired upon exposure to HS, oxidative stress, or other stressors [74–76].

Several pathways related to synaptic transmission were identified for HS time-period 2, including glutamatergic synapse, serotonergic synapse, dopaminergic synapse, GABAergic synapse, cholinergic synapse, and retrograde endocannabinoid signaling. Previous studies have shown that thermal stress is associated with suppressed overall synaptic transmission (especially GABAergic, glutamatergic synapse) and receptor loss [77]. In addition, GABAergic and glutamatergic synapse have been shown to be involved in the synthesis of heat shock proteins (HSP), which contributes to the repair of stress-induced synaptic protein damage and facilitates neuroprotective mechanisms [78, 79]. Cheruiyot et al. [80] showed that the glutamatergic synapse pathway is highly enriched for heat tolerance in Holstein cows [80]. Another pathway, circadian entrainment was identified for HS time-period 2, which is relevant since there is increasing evidence that thermoregulation is controlled by circadian rhythm and that animals with well-entrained circadian rhythms may be better able to cope with HS by optimizing their physiological responses to the stressor [81, 82]. In addition, Li et al. [83] used KEGG and GO enrichment analyses to demonstrate an association between circadian rhythm and heat stress response in Hu sheep.

Disease and development related pathways were mostly enriched for HS time-period 3, with particular emphasis on pathways related to cancer and cardiovascular disease, such as breast cancer and gastric cancer. Heat stress has been shown to activate HSP, which can promote cell proliferation and survival and may be involved in the development of cancer [84, 85]. In addition, as previously mentioned, HS increases the prevalence of cardiovascular diseases, potentially through the pro-inflammatory and pro-atherosclerotic activities of hormones, such as aldosterone [86]. Moreover, during HS time-period 3, sows were observed to suffer from the accumulated HS and produced more heat, leading to the activation of multiple pathways, including thermogenesis, hippo signaling pathway, mTOR signaling pathway, and Wnt signaling pathway. Machado et al. [87] showed that the thermogenesis pathway is related to stress-induced hyperthermia. Thermogenesis can also increase the capacity of endotherms to keep thermal homeostasis under cold stress conditions [88]. Hippo, mTOR, and Wnt signaling pathways are involved in various cell processes, such as cell proliferation, differentiation, and cell death. These pathways also regulate tissue homeostasis in multiple species [89, 90].

The main pathways identified for HS time-period 4 were related to death (nucleotide metabolism, pyrimidine metabolism, and purine metabolism) and hormone functions (e.g., renin secretion). During this HS time-period, sows maintained high body temperatures for several hours and suffered from HS for a longer period. Das et al. [91] showed that increased levels of pyrimidine and purine can activate a tolerance mechanism to protect nucleic acids and ultimately protein synthesis from HS. Stasolla et al. [92] found that alterations in pyrimidine metabolism could be an early signal of apoptosis and could be caused by an increase in endogenous nitric oxide (NO). HS increases the level of reactive oxygen species (ROS) and leads to dysfunction and destruction of cell membranes and finally causes cell death [93]. Both the pyrimidine and purine metabolism pathways have been reported to be enriched in response to HS in buffalo [94], beef cattle [95], and dairy goats [96]. It is not surprising that renin secretion was enriched for HS time-period 4, since animals may undergo severe dehydration during this stage, which can activate the renin–angiotensin–aldosterone system and cause hormonal responses to retain water and maintain mineral homeostasis [97]. The spliceosome pathway was also identified for this HS time-period, which is consistent with the report of Hu et al. [10] who showed that alternative splicing is an important transcriptional mechanism in heat stressed dairy cattle.

### GWAS enrichment along the EG scale

Numerous HS-related pathways were identified based on the uniquely enriched genes for each EG class. Three pathways (other types of O-glycan biosynthesis, Fc gamma R-mediated phagocytosis, and signaling pathways regulating pluripotency of stem cells) were enriched exclusively for EG class 1. These are related to glycan metabolism, immunity, and stem cell. Heat stress induces the synthesis of HSP, which could affect stem cells, including their self-renewal, differentiation, sensitivity to environmental stress, and aging [98].

Five significantly enriched pathways were identified for genes that were uniquely enriched for EG class 2, and their functions were mainly associated with energy (i.e., carbohydrate digestion and absorption, mineral absorption, and bile secretion) and cell proliferation (e.g., oocyte meiosis, cell cycle). Carbohydrate is one of the most important energy sources and it is clear that HS has complex effects on carbohydrate metabolism through increased demands for glucose and changes in insulin signaling and sensitivity [99]. Bile functions are important for fat digestion and metabolism and contribute to growth and the intestine development via the hormones and pheromones that are excreted in bile. Supplementation with bile acids has been shown to reduce the harmful effects of HS in broiler chickens but this has not been studied in cattle or pigs [100]. Heat stress response markedly alters postabsorptive carbohydrate, lipid, and protein metabolism independently of reduced feed intake through coordinated changes in fuel supply and utilization by multiple tissues [99].

Nervous system related pathways (neuroactive ligand-receptor interaction and long-term depression) and nicotinate and nicotinamide metabolism were enriched for genes that were uniquely enriched for EG class 3. The nervous system plays a vital role in controlling and regulating body temperature by continuously monitoring body temperature and initiating appropriate responses, such as vasodilatation, sweating, and reduced metabolic rate, to maintain homeostasis [43]. In ducks, a neuroactive ligand-receptor interaction that is involved in maintaining energy homeostasis during HS has been identified in a previous study [101]. Studies in humans have demonstrated that HS induces mental issues such as chronic depression or chronic anxiety disorders [102, 103]. Nicotinamide provides anti-inflammatory and neuroprotective effects by increasing oxidative phosphorylation, buffering and preventing metabolic stress, and increasing mitochondrial size and motility [104, 105].

Taken together, the results suggest that the genes that have a dynamic function are important in regulating HS across various time-periods or EG classes and in the related pathways for HS in mammals. These findings are of interest for future research towards understanding and managing HS for coping with rising global temperatures. Greater knowledge of the biological basis of response to HS can help farmers and breeders design improved breeding programs and make selection decisions. However, heritability estimates for T_V_ are not available for the whole lactation period because of the data collection scheme implemented in our study. Such results could be useful and contribute to investigating how lactation affects body temperature under HS conditions. Moreover, more potential novel indicators of heat tolerance using body temperature data should be explored. As global warming intensifies, there is an increasing need for breeding more climatically-resilient livestock. Such animals should not only be minimally affected by potential disruptions due to climate changes and farm management but also demonstrate better health, welfare, and improved production efficiency. Thus, the next step is to derive novel indicators of climatic resilience in lactating sows based on variability in Tv and investigate the genetic relationship of these novel traits with production indicators and other routinely recorded traits in swine breeding programs.

## Conclusions and implications

Automatically-recorded vaginal temperature is a promising indicator of HS response for breeding programs due to its moderate heritability and repeatability estimates. The RRM with Legendre orthogonal polynomials (i.e., LEG4) was identified as the best model due to its low BIC value and computational requirements. GxE interactions for T_V_ were identified, indicating that HS could result in re-ranking of breeding values under different climatic challenges or timing of HS. T_V_ is a highly polygenic trait and is controlled by multiple genomic regions of small effects. Twelve, 13, 16, and 10 relevant 10-SNP windows located on more than 10 *Sus scrofa* chromosomes (SSC) were estimated to explain more than 0.25% of the genetic variance for T_V_ across the four respective HS time-periods. For the three respective EG classes, 18, 15, and 14 10-SNP windows were identified for T_V_. Two genomic regions, on SSC10 (59.370–59.998 Mb) and SSC16 (21.548–21.966 Mb), were identified across all HS time-periods and EG classes. Most genomic regions related to T_V_ have dynamic regulatory functions over time and under varying climatic conditions, and specific biological functions were identified for each HS time-period and EG class, such as those related to immunity, metabolism, and hormone. This study provides important insights to conduct genomic selection for improved heat tolerance in pigs, especially during lactation, based on automatically-measured T_V_.

### Supplementary Information

**Additional file 1:** **Figure S1.** Overall schematic representation of the study.

**Additional file 2:** **Figure S2** Patterns of the accuracy of the genomic-estimated breeding values (GEBV) over time based on random regression models (RRM) fitting orthogonal Legendre polynomials (**a**) or B-splines (**b**).

**Additional file 3:** **Table S1.** Candidate genes for the four heat stress (HS) time-periods. **Table S2.** Kyoto Encyclopedia of Genes and Genomes (KEGG) pathways including the candidate genes identified for the four heat stress (HS) time-periods. **Table S3.** Gene Ontology (GO) terms including the candidate genes identified for the four heat stress (HS) time-periods. **Table S4.** Gene Ontology (GO) terms including the candidate genes identified for the three environmental gradient (EG) classes. **Table S5.** Candidate genes for the three environmental gradient (EG) classes.

## Data Availability

All the data supporting the results of this study are included in the article and in the Supplementary Material section. The datasets can be made available for research purposes based upon reasonable request to the corresponding author (Dr. Luiz F. Brito; britol@purdue.edu).
