# Supplementary figures and images for: Longitudinal genomic analyses of automatically-recorded vaginal temperature in lactating sows under heat stress conditions based on random regression models
Source: Genet Sel Evol. 2023 Dec 21;55:95. doi: 10.1186/s12711-023-00868-1 (PMC10734178; doi:10.1186/s12711-023-00868-1)

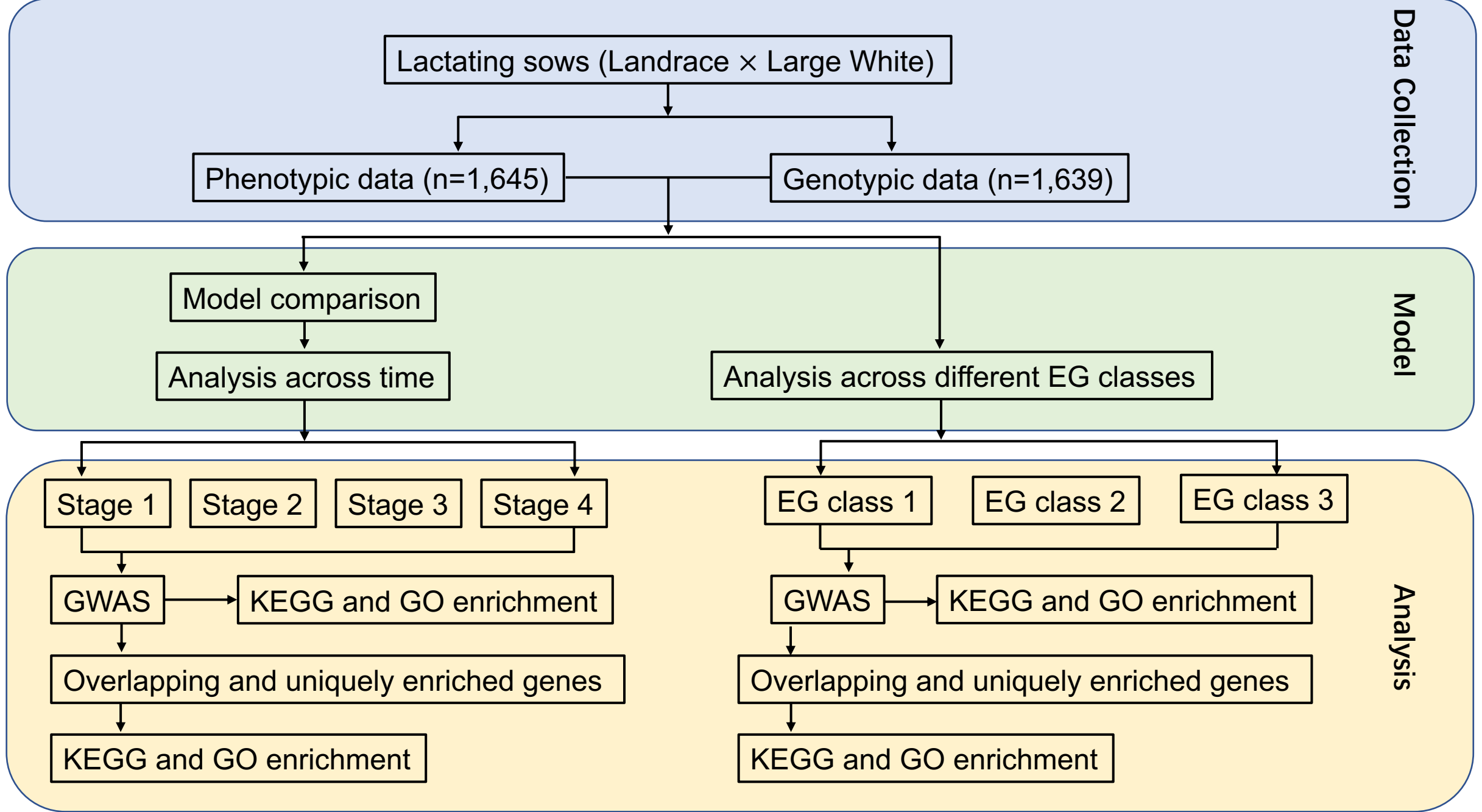

Additional file 1 S1. The overall schematic representation of our study

Supplement: Supplementary file 1 — Additional file 1: Figure S1. Overall schematic representation of the study. [file 12711_2023_868_MOESM1_ESM.pdf]

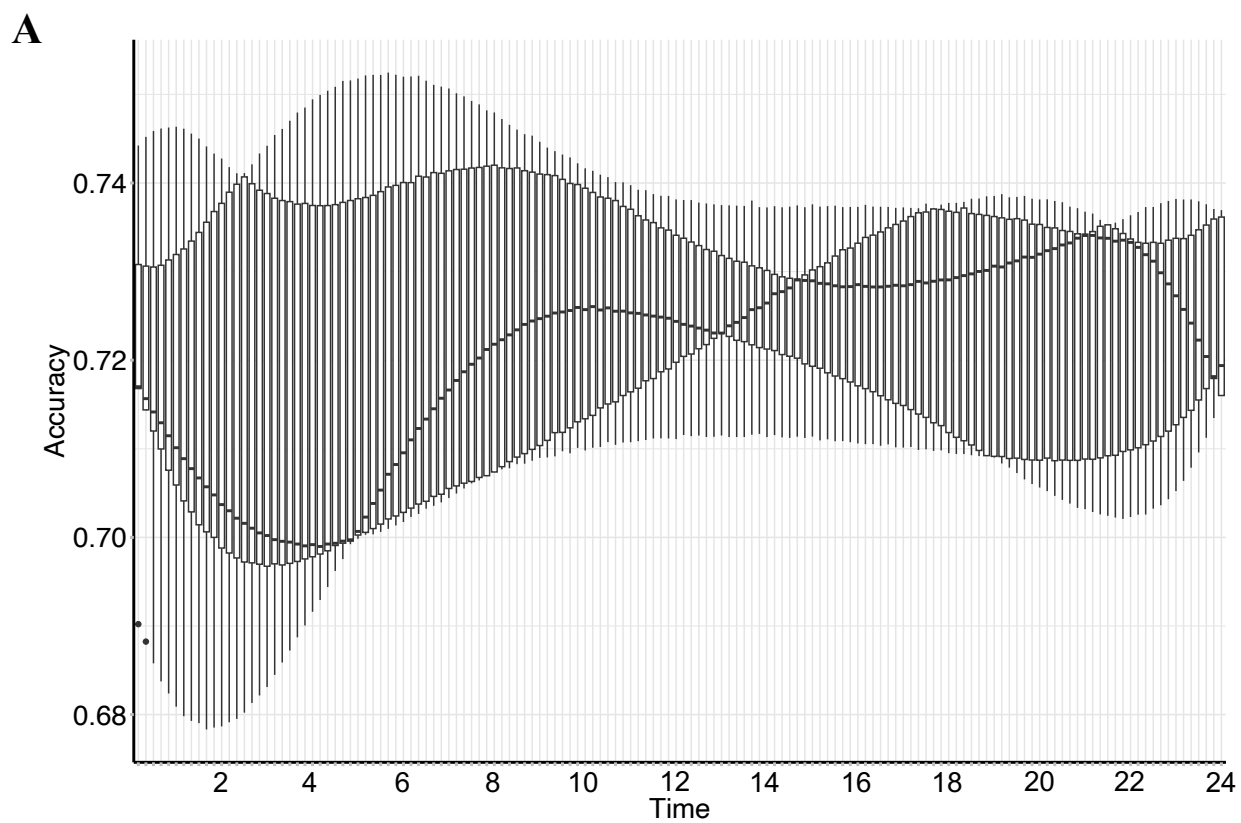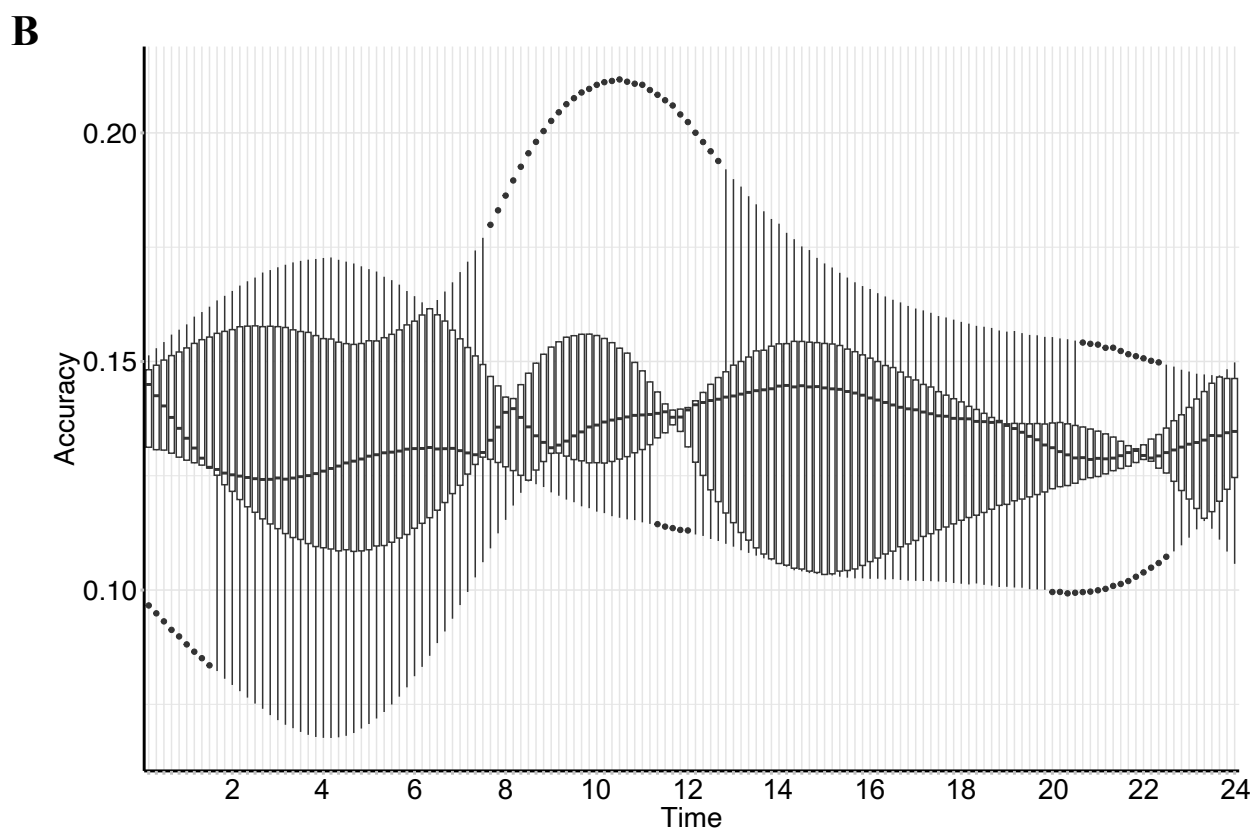

Additional file 2 S2. The accuracy patterns of GEBV over time of A) LEG4 and B) BSQ88

Supplement: Supplementary file 2 — Additional file 2: Figure S2 Patterns of the accuracy of the genomic-estimated breeding values (GEBV) over time based on random regression models (RRM) fitting orthogonal Legendre polynomials (a) or B-splines (b). [file 12711_2023_868_MOESM2_ESM.pdf]
